# EKOS^TM^ in Octogenarians: The Safety and Efficacy of Ultrasound-Accelerated Catheter-Directed Thrombolysis in Elderly Patients with Intermediate-High-Risk Pulmonary Embolism

**DOI:** 10.3390/jcm12072712

**Published:** 2023-04-04

**Authors:** Hani Al-Terki, Abdelrahman Elhakim, Andreas Mügge

**Affiliations:** 1 Cardiology and Rhythmology Department, University Hospital St Josef-Hospital Bochum, Ruhr University Bochum, Gudrunstraße 56, 44791 Bochum, Germany; 2 Cardiology Department, Schoen Hospital, 23730 Neustadt in Holstein, Germany

**Keywords:** EKOS^TM^, pulmonary embolism, octogenarians, USAT

## Abstract

Background: Pulmonary embolism (PE) is a common cardiovascular disease. Elderly patients with acute PE have very high mortality rates. Data concerning the safety and effectiveness of ultrasound-accelerated thrombolysis (USAT) in this age group are lacking. Methods: Nineteen octogenarians with acute pulmonary embolism underwent USAT between August 2020 and February 2023 at two centres in Germany and were retrospectively analysed. The main efficacy measures were the right ventricle to left ventricle diameter (RV/LV) ratio, systolic right ventricle function, and invasive and echocardiographic measured systolic pulmonary artery pressure (sPAP). The main safety measures were in-hospital death and the bleeding rate according to the GUSTO bleeding score. Results: USAT was associated with an improved RV/LV ratio (0.36 ± 0.29, *p* < 0.001), systolic right ventricle function (5.0 ± 3.8, *p* < 0.001), and systolic pulmonary artery pressure (sPAP) at 24 h after therapy (24.2 ± 11.2 mmHg and 19 ± 13.4 mmHg, *p* < 0.001). No in-hospital deaths or bleeding complications occurred. Conclusions: USAT with EKOS^TM^ may be a safe and effective therapeutic option for octogenarians with acute pulmonary embolism.

## 1. Introduction

Acute pulmonary embolism (PE) is the third leading cause of mortality in the Western world [1]. Due to demographic changes, the definition of the ‘elderly’ population in cardiology literature has evolved, initially being people >70 years, before increasing to >75 years [2], and now being >80 years of age [3,4]. In subjects aged ≥80 years with acute PE, the 30-day mortality is estimated to be 18.9% [5].

European guidelines recommend the risk stratification of patients with PE based on clinical symptoms, signs of haemodynamic instability, laboratory findings, and echocardiographic parameters into low-risk, intermediate-low-risk, intermediate-high risk, and high-risk categories. Patients with intermediate-risk PE who deteriorate haemodynamically upon anticoagulation are good candidates for catheter-directed thrombolysis (CDT) [1].

Ultrasound-accelerated thrombolysis (USAT) using EKOS^TM^ (Boston Scientific, Marlborough, MA) is a CDT system that combines low-power ultrasound energy with low-dose thrombolytic agents to achieve clot dissolution. The safety and efficacy of EKOS^TM^ have been investigated in several studies [6,7,8,9]. The mean age in all these studies was 59–63 years. Therefore, the purpose of this study was to assess the clinical outcomes of octogenarians following USAT.

## 2. Methods

Our study population comprised 19 retrospectively evaluated patients (9 males, 10 females, aged 82.9 ± 2.4 years) out of 93 patients who were referred to St. Josef Hospital Bochum and Schoen Clinic Neustadt in Holstein, Germany, with a diagnosis of acute PE and underwent USAT from August 2020 to February 2023.

Confirmation of the diagnosis for all patients was based on contrast-enhanced computed tomography. The evaluation of right ventricle function/strain was carried out with transthoracic echocardiography (TTE) and by measuring cardiac biomarkers (troponin T and NTproBNP). All PE patients were classified according to the ESC guidelines as having low-, intermediate-low-, intermediate-high-, or high-risk PE.

Octogenarians who deteriorated haemodynamically upon anticoagulation therapy were considered suitable candidates for USAT with EKOS^TM^, which was performed in the cardiac catheterization lab within 24 h.

The EKOS^TM^ system involves positioning one (for unilateral PE) or two pulmonary arterial infusion catheters, with one inserted into each main pulmonary artery (PA), under fluoroscopic guidance via percutaneous transvenous access. The procedure was performed with continuous haemodynamic and electrocardiogram (ECG) monitoring. Over 6 h, 6 mg of recombinant-tissue plasminogen activator (rt-PA) was continuously administered at a rate of 1 mg/h per catheter. Heparin was administered concomitantly with doses determined using a hospital-defined nomogram that protocolizes dosing to a target partial thromboplastin of 60 s. After 6 h, the system was stopped, and the infusion catheters were removed, followed by compression of the femoral vein for 4 h.

After USAT with rt-PA, all patients received unfractionated heparin for a further 2 h and were then switched to oral anticoagulants and transferred to a normal ward. Twenty-four hours after therapy, another TTE was conducted to measure tricuspid annular pulmonary systolic excursion (TAPSE), the systolic pulmonary artery pressure (sPAP), RV end-diastolic diameter (RVEDd), and the RV/LV ratio. In addition, creatinine, troponin T, and NTproBNP were monitored for 24 h after the therapy. Vital parameters (respiratory rate, heart rate, and blood pressure) were regularly monitored from admission to transfer to the normal ward.

### 2.1. Efficacy Measures

As compared to baseline, improvements in the measures of the RV/LV ratio and right ventricle function, as assessed by echo and invasively assessed, as well as echo-measured sPAP 24 h after the therapy, were used as substitutes for USAT efficacy.

### 2.2. Safety Measures

The safety outcomes included minor and non-fatal major bleeding according to the GUSTO bleeding score and all-cause mortality during the hospital stay and 30 days after the therapy.

### 2.3. Statistical Analysis

**Figure 1:**
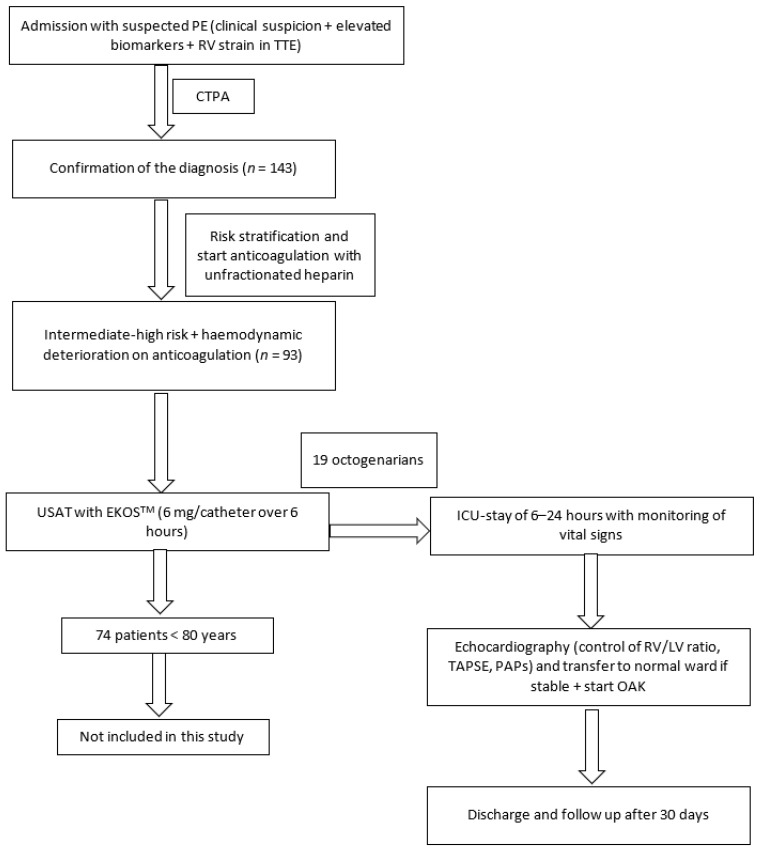
Study flowchart.

Continuous variables were expressed as the mean ± standard deviation, and categorical variables were expressed as percentages. All statistical analyses were performed using SPSS Version 25.0 for Windows. A comparison between variables was performed with the 2-tailed *t*-test. A *p*-value < 0.05 was considered statistically relevant. All statistical analyses were performed by a blinded analyst (Figure 1).

## 3. Results

### 3.1. Study Population

Nineteen patients with intermediate-high-risk PE who underwent USAT with EKOS^TM^ were included in this study and retrospectively analysed. Ten were women (52.6%). The mean age was 82.9 years (SD 2.4), and the mean body mass index (BMI) was 27.7 kg/m^2^ (SD 2.9). Five patients (26.3%) had chronic heart failure, and thirteen (68%) had arterial hypertension. Seven (36.8%) had been previously diagnosed with PE. Nine subjects (47.1%) had a trigger, such as cancer (*n* = 3, 15.7%) or immobility (*n* = 6, 31.4%). The baseline characteristics are summarized in Table 1.

### 3.2. Initial Presentation and Clinical and Echocardiographic Parameters at Admission

The most common primary complaint was dyspnoea (*n* = 15, 78.9%). One patient (5%) had syncope, two (10%) presented with angina pectoris, and one (5%) reported dizziness as the main complaint.

The median heart rate at admission was 92.2 bpm (SD 11.6), the median systolic blood pressure was 131.2 mmHg (SD 33.8), and the median respiratory rate was 22.7 per minute (SD 4.8).

The initial RV/LV ratio measured by TTE was 1.22 (SD 0.19). The median RV/LV ratio measured by CT was 1.37 (SD 0.37). The mean sPAP was 53.13 mmHg (SD 13.8). The mean tricuspid annular plane systolic excursion (TAPSE) was 15.4 mm (SD 4.0).

The median initial creatinine value was 1.12 (SD 0.35), the mean initial NTproBNP was 7465.8 pg/mL (SD 9409) (normal value < 125 pg/mL), and the median haemoglobin value was 11.3 mg/dl (SD 3.0). The median Troponin was as 0.11 pg/mL (SD: 0.13) (reference range at our institution < 0.01 pg/mL), indicating severe cardiac injury. Table 2 summarizes the clinical, chemical laboratory, and echocardiographic parameters at admission.

### 3.3. Procedural Data

Four patients (21.2%) had right-side PE and were treated with just one EKOS^TM^ catheter in the right pulmonary artery. One patient (5.2%) had left-side PE, with the majority (14 patients, 73.6%) having bilateral PE. The median procedural time was 19.1 min (SD 18), and the mean fluoroscopy time was 10.1 min (SD 6.6). Table 3 summarizes the procedural data and post-USAT stay values.

### 3.4. Post-Procedure Data (Clinical Parameters, Echocardiography, and Laboratory)

The RV/LV ratio, sPAP, and TAPSE decreased significantly after therapy. NTproBNP also significantly decreased. Table 4 summarizes the mean values before and after therapy. All control values were recorded at 24 h after therapy.

### 3.5. Complications

There was no device failure in our study. No in-hospital deaths occurred. One subject (5%) had mild access site hematoma, and another patient (5%) had a pseudoaneurysm due to mis-puncture of the A. femoralis. All access-related complications were treated conservatively without the need for surgical intervention.

### 3.6. Follow-Up after 30 Days

The patients were all contacted by their physicians after one month. During the 30-day follow-up period, there were no deaths, recurrent embolisms, or bleeding events.

## 4. Discussion

The main finding of this study is that USAT with EKOS^TM^ represents a safe and efficient therapeutic option for octogenarians with intermediate-high-risk PE who deteriorate haemodynamically upon anticoagulation.

The elderly are the fastest-growing segment of Western populations. Currently, there are over 6 million octogenarians in Germany, a number that is expected to increase in the future [10]. Despite the high prevalence of PE among this group of patients, they have been frequently underrepresented in previous studies. The presence of multiple comorbidities in octogenarians makes diagnosis difficult, which, in turn, leads to misdiagnosis and therefore high mortality, estimated to be as high as 29.5% in some studies [11].

In general, PE patients who deteriorate haemodynamically upon anticoagulation should receive reperfusion therapy [1], but the use of systemic thrombolytics can be difficult in elderly patients due to their high burden of comorbidities, other complications such as bleeding, and, consequently, higher mortality rates. As an alternative to systemic thrombolytics, catheter-directed-treatments should be considered [1].

Zengin et al. found that the use of systemic thrombolytics in octogenarians with intermediate-high- or high-risk PE can significantly reduce in-hospital mortality (18.9% versus 10.5%, *p* = 0.03), but this comes at the expense of overall bleeding complications (21.6% versus 35.1%, *p* < 0.01, for minor bleeding and 6.1% versus 5.5%, *p* = 0.71, for major bleeding) [11]

Meyer et al. found higher extracranial bleeding rates in patients over aged 75 years when compared to younger patients treated with thrombolytic agents. However, this increase was not statistically significant (*p* = 0.09) [12].

We report on the first study of the use of USAT with low-dose thrombolytics in 19 octogenarians with a mean age of 82.9 years. The primary efficacy criteria were achieved for all the patients. There were no major bleeding events or in-hospital deaths. At 30-day follow-up, all the patients were alive. No recurrent embolism or bleeding events occurred.

While the ULTIMA trial and the Seattle II trial did not include any patients over 80 years of age, the decrease in the RV/LV ratio in our cohort (0.36 ± 0.29) is comparable with the results of these studies. The mean difference in echo-measured sPAP (19 mmHg) in our study exceeds that of the Seattle II study (14 mmHg). This may be explained by the fact that Seattle II included more high-risk PE patients than the current study. This explains the higher RV/LV ratio at admission in Seattle II (1.55 versus 1.22 in our study).

An increased RV/LV ratio is a reproducible and well-validated tool for identifying PE patients at high risk of adverse outcomes and increased 30-day mortality [13]. The Seattle II study proposed the idea that further studies should investigate the effects of USAT on haemodynamic parameters. In our study, we showed that USAT can significantly reduce the heart rate (12.1 ± 11.5 bpm, *p* < 0.001) and the respiratory rate (5.0 ± 3.6, *p* < 0.001) but not the systolic blood pressure (0.89 ± 34.5 mmHg, *p* = 0.9), which confirms that our cohort represents haemodynamically stable patients. It remains unclear whether or not the use of USAT is generally efficient for patients who are haemodynamically unstable at admission.

Zengin et al. reported an in-hospital mortality of 18.9% in octogenarians with acute PE [11]. There were no in-hospital mortality or bleeding events in our study, which could mean that USAT may be an efficient and safe therapeutic option for this group. It is generally difficult to draw firm conclusions about the long-term efficacy of a therapy in very elderly patients, since the long-term survival is limited in this group due to underlying comorbidities rather than PE itself [14]. Elderly patients with acute PE should not be excluded from new PE interventional options due to their advanced age. Taking into consideration the known bleeding risks of systemic thrombolytics in octogenarians, we believe that USAT is a highly suitable therapeutic option.

## 5. Conclusions

Our study demonstrates that reperfusion therapy with USAT may reduce the known level of in-hospital mortality in octogenarians with acute PE without increasing the occurrence of bleeding complications.

## 6. Limitations

The main limitations of our study were the small sample size and the lack of a control group, randomization, and echocardiographic follow-up. Large, randomized studies are needed to validate our findings and to determine the optimal dosage needed to achieve the best outcomes.

## Figures and Tables

**Table 1:** Baseline characteristics. BMI: body mass index, OSAS: obstructive sleep apnoea syndrome, COPD: chronic obstructive pulmonary disease, CHF: chronic heart failure, PE: pulmonary embolism, and SD: standard deviation.

	Patient Characteristics	*n* = 19
Characteristic	Age, mean (SD)	82.9 (2.4)
	Sex (female)	10 (52.6%)
	BMI, mean (SD)	27.7 (2.9)
Comorbidities	Arterial hypertension, *n* (%)	13 (68%)
	Diabetes mellitus, *n* (%)	1 (5%)
	CHF, *n* (%)	5 (26.3%)
	Coronary artery disease, *n* (%)	3 (15.7%)
	Cancer, *n* (%)	3 (15.7%)
	Previous PE, *n* (%)	7 (36.8%)
	Immobility, *n* (%)	6 (31.4%)
	COPD, *n* (%)	2 (10.5%)
	Asthma, *n* (%)	1 (5%)
	COVID, *n* (%)	1 (5%)
	Lung emphysema, *n* (%)	2 (10.5%)
	OSAS, *n* (%)	4 (21%)

**Table 2:** Clinical, chemical laboratory, and echocardiographic parameters at admission. CPR: cardiopulmonary resuscitation, RVEDd: right ventricle end-diastolic diameter, LVEDd: left ventricle end-diastolic diameter, sPAP: pulmonary artery pressure, TAPSE: tricuspid annular plane systolic excursion, SD: standard deviation, PESI: pulmonary embolism severity index.

	Patient Characteristics	*n* = 19
Initial presentation	Dyspnoea, *n* (%)	15 (78.9%)
	Syncope, *n* (%	1 (5%)
	CPR, *n* (%)	0 (0%)
	Angina pectoris, *n* (%)	2 (10%)
	Dizziness	1 (5%)
Clinical parameters at admission	Heart rate, mean (SD)	92.2 bpm (11.6)
	Systolic pressure, mean (SD)	131.2 mmHg (33.8)
	Respiratory rate, mean (SD)	22.7 (4.8)
	Oxygen saturation, mean (SD)	89.5% (6.0)
	PESI score, mean (SD)	124 (26.3)
Echocardiography parameters at admission	RVEDd, mean (SD)	45.4 mm (5.9)
	LVEDd, mean (SD)	37.9 mm (5.4)
	sPAP, mean (SD)	53.1 mmHg (13.8)
	TAPSE, mean (SD)	15.4 mm (4.0)
	RV/LV ratio, mean (SD)	1.22 (0.19)
Laboratory chemical values	Creatinine, mean (SD)	1.1 mg/dL (0.35)
	NTproBNP, mean (SD)	7465.8 pg/mL (9409)
	Troponin T, mean (SD)	0.11 pg/mL (0.13)
	Haemoglobin, mean (SD)	11.3 mg/dL (3.0)
	Lactate, mean (SD)	2.2 mmol/L (2.7)

**Table 3:** Procedural data and postintervention stay. ICU: intensive care unit, SD: standard deviation.

		*n* = 19
Procedural data	Procedure time (min), median (SD)	19.1 (18)
	Contrast (mL), median (SD)	14.7 mL (27.7)
	Fluoroscopy dose (Gy), median (SD)	2617 cGY/cm^2^ (5286)
	Fluoroscopy time (min), median (SD)	10.1 min (6.6)
Post-procedure stay	ICU stay (days), median (SD)	3.6 (9.3)
	Total stay (days), median (SD)	11.8 (12)

**Table 4:** Mean values before and after therapy. USAT: ultrasound-accelerated thrombolysis, CI: confidence interval, TAPSE: tricuspid annular plane systolic excursion, sPAP: systolic pulmonary artery pressure, RVEDd: right ventricle end-diastolic diameter, LVEDd: left ventricle end-diastolic diameter, RV/LV ratio: right ventricle to left ventricle diameter ratio, NTproBNP: N-terminal pro-brain natriuretic peptide.

	Before USAT	After USAT	Mean Absolute Change, 95% CI	t	*p*-Value
Heart rate	92.2 ± 11.6 bpm	80.0 ± 9.3	12.15 (6.5–17.7)	4.5	<0.001
Respiratory rate	22.7 ± 4.8	17.6 ± 4.3	5.0 (3.0–7.1)	5.3	<0.001
Systolic blood pressure	131.2 ± 23.8 mmHg	130.3 ± 26.8	0.89 (–15.7–17.5)	0.11	0.9
TAPSE	15.47 ± 4.0 mm	20.5 ± 3.6 mm	−5.0 (−7.2 to −2.9)	−5.0	<0.001
sPAP (by echo)	53.13 ± 13.8 mmHg	34.13 ± 9.3 mmHg	19.0 (11.5–26.4)	5.4	<0.001
TAPSE/sPAP	0.31 ± 0.14	0.65 ± 0.20	−0.34 (–0.47 to −0.21)	−5.5	<0.001
RVEDd	45.47 ± 5.99 mm	37.2 ± 3.7 mm	8.2 (5.4–11.0)	6.3	<0.001
LVEDd	37.94 ± 5.4 mm	41.3 ± 5.4 mm	−3.3 (−6.4 to −0.2)	−2.3	0.03
RV/LV ratio	1.22 ± 0.19	0.85 ± 0.26	0.36 (0.21–0.52)	5.0	<0.001
sPAP (invasive measure)	56.7 ± 9.1 mmHg	32.5 ± 6.8 mmHg	24.2 (14.8–33.6)	6.09	<0.001
NTproBNP	7465.8 ± 9409.35 pg/mL	5623.4 ± 7781.21 pg/mL	1842 (318–3366)	2.66	0.02

## Data Availability

Where data is unavailable due to privacy and ethical restrictions.

## Abbreviations

Body mass index	BMI
Cardiopulmonary resuscitation	CPR
Catheter-directed thrombolysis	CDT
Chronic heart failure	CHF
Chronic obstructive lung disease	COPD
Computed tomography	CT
Computed tomography pulmonary angiography	CTPA
Electrocardiogram	ECG
European Society of Cardiology	ESC
Left ventricle	LV
N-terminal pro-brain natriuretic peptide	NTproBNP
Obstructive sleep apnoea syndrome	OSAS
Pulmonary embolism	PE
Recombinant tissue plasminogen activator	rt-PA
Right ventricle	RV
Standard deviation	SD
Systolic pulmonary artery pressure	sPAP
Transthoracic echocardiography	TTE
Tricuspid annular plane systolic excursion	TAPSE
Ultrasound-accelerated thrombolysis	USAT
